# A New Two-Dimensional Map with Hidden Attractors

**DOI:** 10.3390/e20050322

**Published:** 2018-04-27

**Authors:** Chuanfu Wang, Qun Ding

**Affiliations:** Electronic Engineering College, Heilongjiang University, Harbin 150080, China

**Keywords:** hidden attractors, fixed point, stability

## Abstract

The investigations of hidden attractors are mainly in continuous-time dynamic systems, and there are a few investigations of hidden attractors in discrete-time dynamic systems. The classical chaotic attractors of the Logistic map, Tent map, Henon map, Arnold’s cat map, and other widely-known chaotic attractors are those excited from unstable fixed points. In this paper, the hidden dynamics of a new two-dimensional map inspired by Arnold’s cat map is investigated, and the existence of fixed points and their stabilities are studied in detail.

## 1. Introduction

The investigations of the chaotic system were greatly encouraged by the discovery of the Lorenz system [1]. The Lorenz system is one of the most wildly-studied continuous-time dynamic systems, and other classical continuous-time dynamic systems include the Rössler system, Chua system, Chen system, Lü system, and Sprott system [2,3,4,5,6]. Most attractors of those classical continuous-time dynamic systems are excited from unstable equilibria. However, hidden attractors imply the basin of attraction does not contain neighborhoods of equilibria [7]. For finding hidden attractors, a lot of systems improved when classical continuous-time dynamic systems were proposed [8,9,10,11,12,13,14,15,16,17,18,19]. These investigations of hidden attractors can be classified by the number and stability of equilibria, such as no equilibrium, finite stable equilibria, and infinite stable equilibria. In 2010, a special analytical-numerical algorithm of finding hidden attractors in Chua system was proposed [20]. The special algorithm can find the accuracy initial values that lead to hidden attractors and has promoted the development of finding hidden attractors in continuous-time dynamic systems [21,22,23,24]. However, most investigations of hidden attractors are mainly in continuous-time dynamic systems, such as the Chen system, Sprott system, Chua system, and Lü system [15,16,17,18,19,20,21,22,23,24]. There are only a few of investigations of hidden attractors in discrete-time systems.

The classical chaotic maps include the Logistic map, Tent map, Henon map, and Arnold’s cat map [25,26,27,28]. In line with continuous-time dynamic systems, these hidden attractors in the classical chaotic maps are also excited from unstable fixed points. In 2016, Jafari et al. studied a new one-dimensional chaotic map with no fixed point inspired by Logistic map, and its bifurcation and period doubling were introduced [29]. At the same year, Jiang et al. performed a search to find hidden attractors in a new two-dimensional chaotic map inspired by the Henon map and analyzed several different cases on fixed points, such as no fixed point, single fixed point, and two fixed points [30].

In this paper, a new two-dimensional chaotic map inspired by Arnold’s cat map is proposed. For the limitation in the form of the Arnold’s cat map, the number of fixed points in the new two-dimensional chaotic map is not larger than two, and the fixed points are closely related to the Lyapunov exponents. Due to the restriction of the form of the Arnold’s cat map, the new two-dimensional chaotic attractor can only appear in the case of no fixed point. Thus, our concern focuses on the case of no fixed point. The paper is arranged as follows: Section 2 describes the Arnold’s cat map and shows its chaotic attractor. Section 3 analyzes the stability of the equilibria in the new two-dimensional map. Section 4 demonstrates the digitalization and hardware implementation of the new two-dimensional map. The complexity of output time series is tested by approximate entropy in Section 5, and Section 6 summarizes the conclusions of this paper.

## 2. Arnold’s Cat Map

Arnold’s cat map, also known as cat chaotic map, is a chaotic map of repeated folding and stretching in a limited area and is wildly used in multimedia chaotic encryption [28]. Arnold’s cat map is a two-dimensional chaotic map and defined as

(1) $$\left[\begin{array}{c} x(n+1)\\ y(n+1) \end{array}\right]\;=\;\left[\begin{array}{cc} 1 & 1\\ 1 & 2 \end{array}\right]\left[\begin{array}{c} x(n)\\ y(n) \end{array}\right](\mathrm{mod}1)$$

Equation (1) can be transformed into Equation (2) for calculating the fixed points.

(2) $$\{\begin{array}{c} x(n)\;=\;x(n)\;+\;y(n)(\mathrm{mod}1)\\ y(n)\;=\;x(n)\;+\;2y(n)(\mathrm{mod}1) \end{array}$$

**Figure 1:**
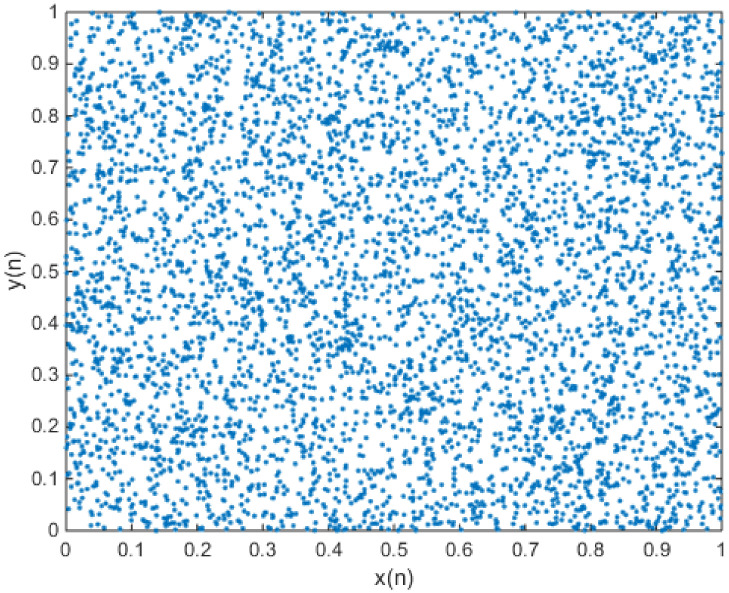
When x(0) = 0.7 and y(0) = 0.6, this represents the phase diagram of Arnold’s cat map.

Equation (2) always has a fixed point, because it is composed of homogeneous linear equations. The fixed point of the Arnold’s cat map is $\{\begin{array}{l} x^{*}\;=\;0\\ y^{*}\;=\;0 \end{array}$. The Jacobian matrix of Arnold’s cat map is $\left[\begin{array}{cc} 1 & 1\\ 1 & 2 \end{array}\right]$, and the two eigenvalues are calculated as $Eig_{1}\;=\;\frac{3\;+\;\sqrt{5}}{2}\;\approx \;2.618$ and $Eig_{2}\;=\;\frac{3\;-\;\sqrt{5}}{2}\;\approx \;0.382$. Therefore, the fixed point (0,0) is an unstable fixed point. It is a saddle point, because there is a positive Lyapunov exponent and a negative Lyapunov exponent. For a positive Lyapunov exponent, Arnold’s cat map is a two-dimensional chaotic map. In Arnold’s cat map, two eigenvalues of the Jacobian matrix are associated, respectively, to an expanding and a contracting eigenspace, which are also the stable and unstable manifolds [31]. The phase diagram of Arnold’s cat map is shown in Figure 1.

The generalized Arnold’s cat map is defined as

(3) $$\left[\begin{array}{c} x(n\;+\;1)\\ y(n\;+\;1) \end{array}\right]\;=\;\left[\begin{array}{cc} 1 & \;a\\ b & \;ab\;+\;1 \end{array}\right]\left[\begin{array}{c} x(n)\\ y(n) \end{array}\right](\mathrm{mod}1).$$

The fixed point of the generalized Arnold’s cat map also is $\{\begin{array}{l} x^{*}\;=\;0\\ y^{*}\;=\;0 \end{array}$. The Jacobian matrix of the generalized Arnold’s cat map is $\left[\begin{array}{cc} 1 & \;a\\ b & \;ab\;+\;1 \end{array}\right]$, and two eigenvalues are $\lambda_{1}\;=\;1\;+\;\frac{ab\;+\;\sqrt{{\left(ab\;+\;2\right)}^{2}\;-\;4}}{2}\;>\;1$ and $\lambda_{2}\;=\;1\;+\;\frac{ab\;-\;\sqrt{{\left(ab\;+\;2\right)}^{2}\;-\;4}}{2}\;<\;1$. Therefore, the fixed point (0,0) also is an addle point. The generalized Arnold’s cat map also is chaotic map, because it has one positive Lyapunov exponent. Despite Arnold’s cat map or the generalized Arnold’s cat map, they always have unstable saddle pint (0,0).

## 3. A New Two-Dimensional Chaotic Map without Fixed Points

In this paper, a new two-dimensional chaotic map inspired by Arnold’s cat map is proposed. It is defined as

(4) $$\left[\begin{array}{c} x(n\;+\;1)\\ y(n\;+\;1) \end{array}\right]\;=\;\left[\begin{array}{cc} a & \;b\\ c & \;d \end{array}\right]\left[\begin{array}{c} x(n)\\ y(n) \end{array}\right]\;+\;\left[\begin{array}{c} e\\ f \end{array}\right](\mathrm{mod}1).$$

in which a = kc + 1, b = k(d − 1), e ≠ kf ≠ 0, e∈(0,1), f∈(0,1), and k ≠ 0. For calculating the fixed point, Equation (4) can be transformed into two-dimensional equations.

(5) $$\{\begin{array}{l} x(n)\;=\;(kc\;+\;1)x(n)\;+\;k(d\;-\;1)y(n)\;+\;e(\mathrm{mod}1)\\ y(n)\;=\;cx(n)\;+\;dy(n)\;+\;f(\mathrm{mod}1) \end{array}$$

Equation (5) can be transformed into nonhomogeneous linear equations by collecting the like terms.

(6) $$\{\begin{array}{l} kcx(n)\;+\;k(d\;-\;1)y(n)\;=\;-e(\mathrm{mod}1)\\ cx(n)\;+\;(d\;-\;1)y(n)\;=\;-f(\mathrm{mod}1) \end{array}$$

There is no solution to the nonhomogeneous linear equations. Thus, the map (4) has no fixed point. However, the coefficients should be further limited for obtaining hidden chaotic attractors. The Jacobian matrix of the map (4) is

(7) $$J_{1}\;=\;\left[\begin{array}{cc} kc\;+\;1 & \;kd\;-\;k\\ c & \;d \end{array}\right].$$

**Figure 2:**
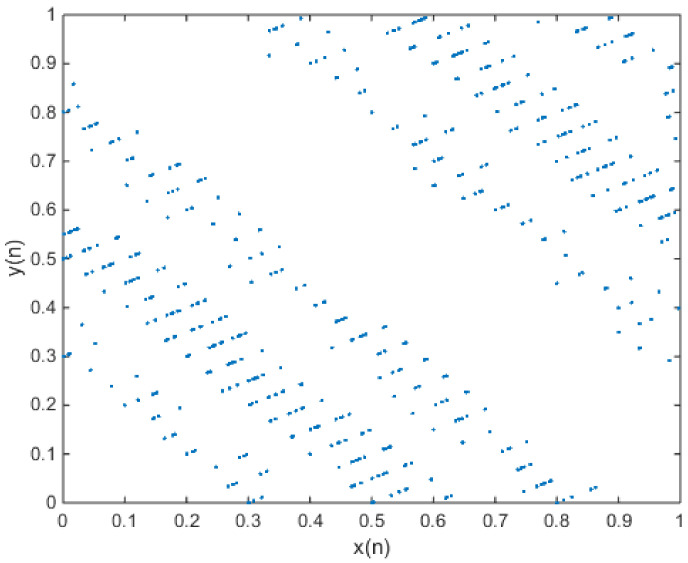
When x(0) = 0.7 and y(0) = 0.6, the phase diagram of the new 2-D map with a = 3, b = 2, c = 1, d = 2, e = 0.1, and f = 0.2.

**Figure 3:**
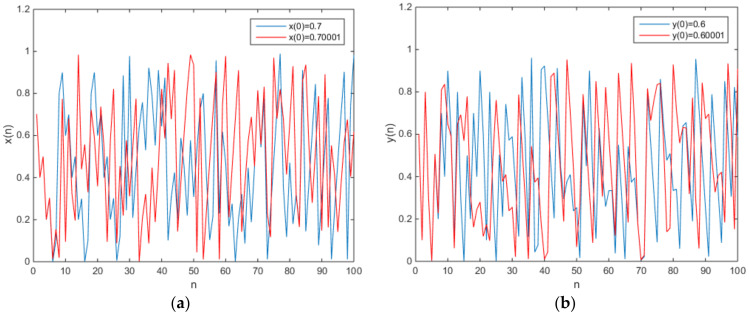
The plot of the output time series (**a**) x(n), (**b**) y(n).

The characteristic equation of the matrix $J_{1}$ is calculated as

(8) $$\det(\lambda I\;-\;J_{1})\;=\;\lambda^{2}\;-\;tr(J_{1})\lambda \;+\;\det(J_{1})\;=\;0.$$

in which $\det（J_{1})\;=\;d\;+\;ck$ is the determinant of matrix $J_{1}$ and $tr(J_{1})\;=\;kc\;+\;1\;+\;d$ is the trace of matrix $J_{1}$. The characteristic equation of the matrix $J_{1}$ is a quadratic function. The roots of the Equation (8) are

(9) $$\lambda_{1,2}\;=\;\frac{kc\;+\;1\;+\;d\;\pm \;(kc\;+\;d\;-\;1)}{2}$$

in which $\lambda_{1}\;=\;1$ and $\lambda_{2}\;=\;kc\;+\;d$. The two corresponding Lyapunov exponents are $LE_{1}\;=\;\ln\left|\lambda_{1}\right|\;=\;0$ and $LE_{2}\;=\;\ln\left|kc\;+\;d\right|$. The non-chaos fixed point attractors have negative Lyapunov exponents. The non-chaos periodic or limit cycle attractors have non-positive Lyapunov exponents. The chaotic attractors have positive Lyapunov exponents. Therefore, the chaotic system exists at least a positive Lyapunov exponent. For obtaining hidden chaotic attractors, the second eigenvalue $\lambda_{2}\;=\;kc\;+\;d$ should be larger than 1. For example, the coefficients are set as c = 1, d = 2, and k = 2, and |kc + d| = 4 > 1. Combining with the map (4), the new two-dimensional map with no fixed point is defined as

(10) $$\left[\begin{array}{c} x(n\;+\;1)\\ y(n\;+\;1) \end{array}\right]\;=\;\left[\begin{array}{cc} 3 & \;2\\ 1 & \;2 \end{array}\right]\left[\begin{array}{c} x(n)\\ y(n) \end{array}\right]\;+\;\left[\begin{array}{c} 0.1\\ 0.2 \end{array}\right](\mathrm{mod}1).$$

in which e = 0.1 and f = 0.2. The phase diagram of attractors is shown in Figure 2, and the plot of the output time series is shown in Figure 3.

The new two-dimensional map (10) is a chaotic map, because it has a positive Lyapunov exponent.

**Figure 4:**
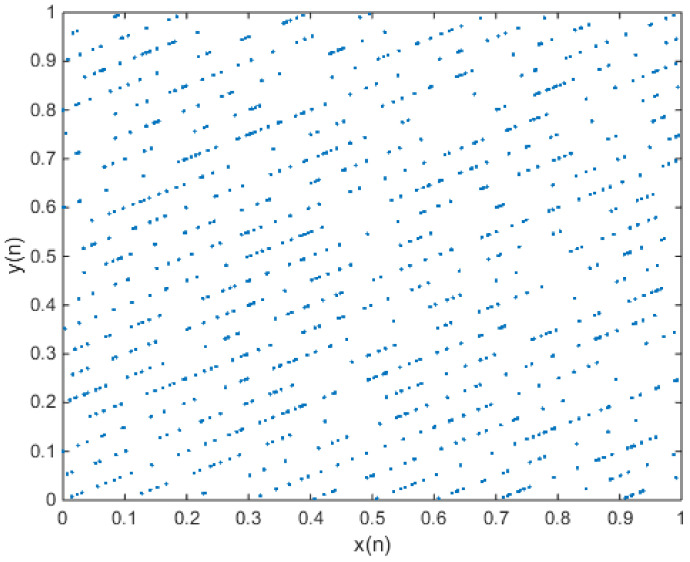
When x(0) = 0.7 and y(0) = 0.6, the phase diagram of the new 2-D map with a = 3.2, b = 2, c = 1.1, d = 2, e = 0.1, and f = 0.2.

**Figure 5:**
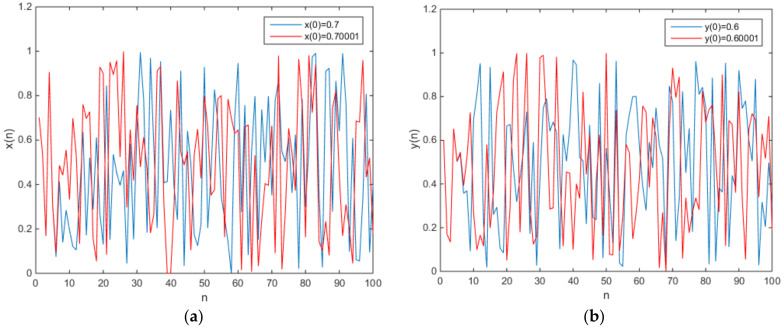
The plot of the output time series (**a**) x(n), (**b**) y(n).

From Equation (9), the chaotic behavior in the map (4) is dependent only on the coefficients c, d, and k, and the second eigenvalue of the Jacobian matrix $J_{1}$ is the simple combination of c, d, and k. The Lyapunov exponent can be changed with different parameters c, d, and k. When c = 1.1, d = 2, k = 2, and t = 4.2 > 1. Combining with the map (4), a new two-dimensional map without fixed points is defined as

(11) $$\left[\begin{array}{c} x(n\;+\;1)\\ y(n\;+\;1) \end{array}\right]\;=\;\left[\begin{array}{cc} 3.2 & \;2\\ 1.1 & \;2 \end{array}\right]\left[\begin{array}{c} x(n)\\ y(n) \end{array}\right]\;+\;\left[\begin{array}{c} 0.1\\ 0.2 \end{array}\right](\mathrm{mod}1).$$

in which e = 0.1 and f = 0.2. The phase diagram of the chaotic attractors is shown in Figure 4, and the plot of the output time series is shown in Figure 5.

**Figure 6:**
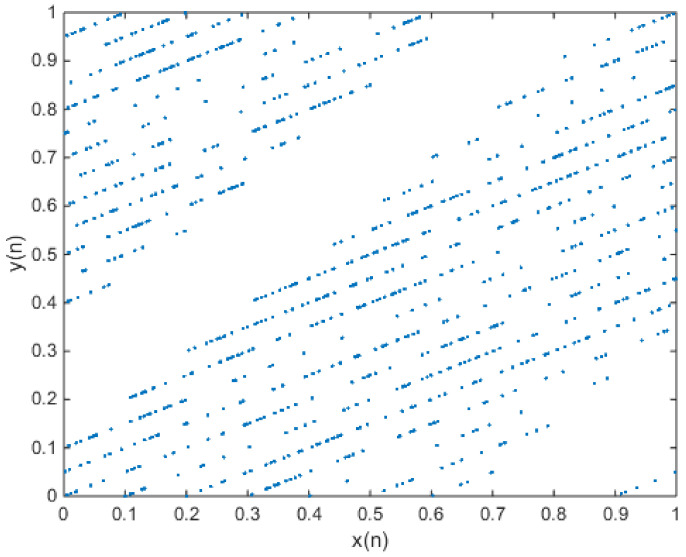
When x(0) = 0.7 and y(0) = 0.6, the phase diagram of the new 2-D map with a = 0.5, b = 2, c = −0.25, d = 2, e = 0.1, and f = 0.2.

**Figure 7:**
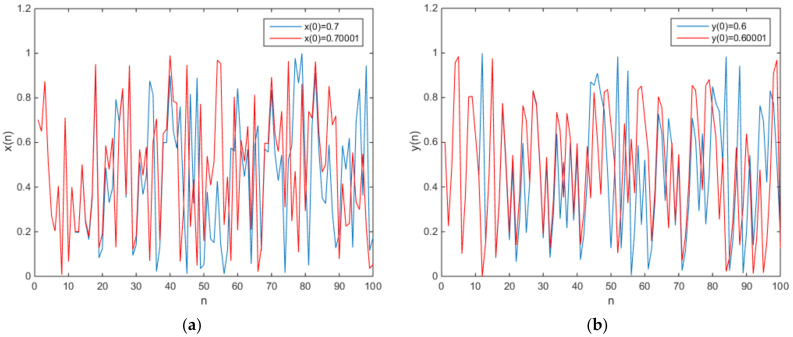
The plot of the output time series (**a**) x(n), (**b**) y(n).

As can be observed from the plots of the time series, it is obvious that the map (11) has initial value sensitivity, randomness, and so on. For no fixed point in the map (11), the map (11) is a chaotic map and has a hidden chaotic attractor. As can be seen from Figure 1, the chaotic attractor of the map (11) is not similar to that of Arnold’s cat map. When c = −0.25, d = 2, k = 2, and t = 1.5 > 1. Combining with the map (4), a new two-dimensional map is defined as

(12) $$\left[\begin{array}{c} x(n\;+\;1)\\ y(n\;+\;1) \end{array}\right]\;=\;\left[\begin{array}{cc} 0.5 & \;2\\ -0.25 & \;2 \end{array}\right]\left[\begin{array}{c} x(n)\\ y(n) \end{array}\right]\;+\;\left[\begin{array}{c} 0.1\\ 0.2 \end{array}\right](\mathrm{mod}1).$$

in which e = 0.1 and f = 0.2. The phase diagram of the chaotic attractors is shown in Figure 6, and the plot of the output time series is shown in Figure 7.

Compared with Figure 1, the phase diagram of the map (12) is dissimilar to the Figure 2 and Figure 4. When initial value is changed, x(n) has the same output time series in the first 20 iterations, and y(n) has the same output time series in the first 10 iterations.

## 4. Digitalization and Hardware Implementation

For the digitalization of information, chaotic systems need to be digitized before they are used [32,33]. The new two-dimensional chaotic map can be digitalized in two ways: one is the floating-point representation, the other is fixed-point representation. According to IEEE 754-2008 [34], floating-point is divided into single precision and double precision. The form of floating-point is shown in Table 1.

The hardware consumptions of the fixed-point representation are not very high, because it includes sign bit and fraction bits. However, there is no standard form in fixed-point representation. Compared with floating-point computing, fixed-point computing is faster, and hardware implementation is smaller [35]. For a better performance in hardware implementation, the new two-dimensional map is represented by fixed-point in this paper. Since x(n) and y(n) are decimal numbers, they are more easily represented by fixed-point. Therefore, x(n) and y(n) are changed from decimals to integers. The digitized N bits of decimal β can be represented as

(13) $$\tilde{\beta }\;=\;\left\lfloor 2^{N}\;\beta \;\right\rfloor 2^{-N}.$$

in which $\tilde{\beta }$ is an N bits approximation of β. The digitized N bits of map φ(β) = λβmod1 can be written as $\tilde{\varphi }(\tilde{\beta })\;=\;\left\lfloor 2^{N}\;\lambda \tilde{\beta }\mathrm{mod}2^{N}\right\rfloor 2^{-N}$, and the digitized N bits of map (4) can be written as

(14) $$\{\begin{array}{c} \tilde{x}(n\;+\;1)\;=\;\left\lfloor 2^{N}\;a\tilde{x}(n)\;+\;2^{N}\;b\tilde{y}(n)\;+\;2^{N}\;e\mathrm{mod}2^{N}\right\rfloor 2^{-N}\\ \tilde{y}(n\;+\;1)\;=\;\left\lfloor 2^{N}\;c\tilde{x}(n)\;+\;2^{N}\;d\tilde{y}(n)\;+\;2^{N}\;f\mathrm{mod}2^{N}\right\rfloor 2^{-N} \end{array}.$$

Multiplying both sides by $2^{N}$, map (14) can be represented as

(15) $$\{\begin{array}{c} 2^{N}\tilde{x}(n\;+\;1)\;=\;\left\lfloor 2^{N}\;a\tilde{x}(n)\;+\;2^{N}\;b\tilde{y}(n)\;+\;2^{N}\;e\mathrm{mod}2^{N}\right\rfloor \\ 2^{N}\tilde{y}(n\;+\;1)\;=\;\left\lfloor 2^{N}\;c\tilde{x}(n)\;+\;2^{N}\;d\tilde{y}(n)\;+\;2^{N}\;f\mathrm{mod}2^{N}\right\rfloor \end{array}.$$

**Figure 8:**
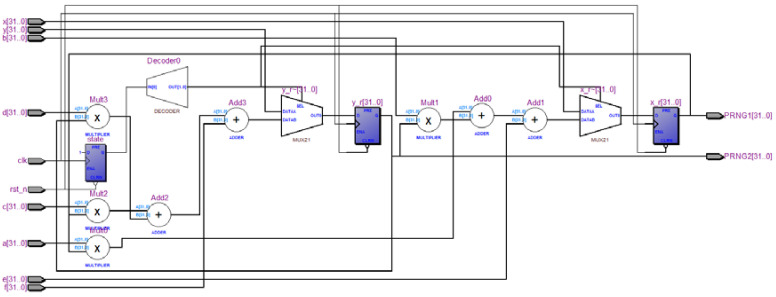
The block diagram of the hardware implementation by FPGA.

Denote $2^{N}\tilde{x}(n)$, $2^{N}\tilde{y}(n)$, $2^{N}e$, and $2^{N}f$ by x(n), y(n), e, and f, respectively. The digitized N bits of map (15) can be written as

(16) $$\left[\begin{array}{c} x(n\;+\;1)\\ y(n\;+\;1) \end{array}\right]\;=\;\left\lfloor \left[\begin{array}{cc} a & \;b\\ c & \;d \end{array}\right]\left[\begin{array}{c} x(n)\\ y(n) \end{array}\right]\;+\;\left[\begin{array}{c} e\\ f \end{array}\right]\right\rfloor (\mathrm{mod}2^{N}),$$

in which x(n) and y(n) is in the interval $\left[0,2^{N}\;-\;1\right]$, N presents the length of finite precision, x(n) and y(n) is represented by N bits. When N = 32, the hardware implementation by FPGA is shown in Figure 8.

## 5. The Analysis of Complexity

The approximate entropy algorithm is proposed from the angle of measuring the complexity of time series [36,37,38,39]. The main idea of the approximate entropy algorithm is using a non-negative value to quantify the complexity and irregularity of the time series, and the value increases with the increase of sequence complexity. The calculation process of the approximate entropy is shown as follows:

1 Suppose the initial data is the sequence x(1), x(2), …x(N), and then divide them into m-dimensional vectors
  (17) X(i) = [x(i),x(i + 1),...,x(i + m − 1)],
  in which i = 1, 2, 3...N − m + 1.
2 The distance between x(i) and x(j) is defined as
  (18) $$d(i,j)\;=\;\underset{k=1-m-1}{\max}[\left|x(i\;+\;k)\;-\;x(j\;+\;k)\right|].$$
3 Setting a threshold value r(r > 0), for each i, we can obtain the statistics of d(i,j).
  (19) $$C_{i}{}^{m}(r)\;=\;\frac{1}{N\;-\;m\;+\;1}Sum\{d(i,j)\;<\;r\}$$
4 The mean of logarithm of $C_{i}{}^{m}(r)$ is written as $\phi^{m}(r)$ and can be calculated by
  (20) $$\phi^{m}(r)\;=\;\frac{1}{N\;-\;m\;+\;1}\sum_{i=1}^{N-m+1}\ln C_{i}{}^{m}(r)$$
5 Changing dimension and repeating step 1 to step 4, we can obtain the approximate entropy
  (21) $$ApEn(m,r)\;=\;\underset{N\to \infty }{\lim}[\phi^{m}(r)\;-\;\phi^{m+1}(r)]$$

However, in practical terms, the length of the data sequence is bounded. Therefore, the approximate entropy algorithm is changed into

(22) $$ApEn(m,r,N)\;=\;[\phi^{m}(r)\;-\;\phi^{m+1}(r)]$$

Pincus found that there exists a minimal dependency between ApEn and N when m = 2 and r∈[0.1SD(x),0.2SD(x)] [36]. SD(x) is the standard deviation of x. The complexity of the output time series of Arnold’s cat map and new two-dimensional chaotic maps are tested by approximate entropy algorithm, and the consequence show that the output time series of Equation (11) has a higher complexity. The specific results are shown in Table 2.

## 6. Conclusions

In this paper, the hidden attractors for the two-dimensional chaotic map are studied, and the existence of fixed points and their stability are considered. Due to the restriction of the form of the Arnold’s cat map, the new two-dimensional chaotic attractor can only appear in the case of no fixed point. The selection of coefficients directly affects whether the two-dimensional map has chaotic behavior, because the eigenvalue of the Jacobian matrix of the new two-dimensional chaotic map is a simple combination of the coefficients. Three concrete examples are given to illustrate the relationship between the coefficients and the chaotic behavior. The different coefficients can not only determine the chaotic behavior of the new two-dimensional chaotic map but also affect the shape of the chaotic attractor.

## Figures and Tables

**Table 1:** Floating-point representation.

Floating-Point	Sign	Exponent	Fraction
Single precision	1 bit	8 bits	23 bits
Double precision	1 bit	11 bits	52 bits

**Table 2:** Approximate entropy test.

Chaotic Map	Time Series	*m*	*r* = 0.15*SD*	*N*	ApEn
Equation (1)	x(n)	2	0.0435	2000	0.9787
	y(n)	2	0.0436	2000	0.9963
Equation (10)	x(n)	2	0.0426	2000	0.7591
	y(n)	2	0.0437	2000	0.8841
Equation (11)	x(n)	2	0.0436	2000	1.4171
	y(n)	2	0.0434	2000	1.2831
Equation (12)	x(n)	2	0.0438	2000	0.5433
	y(n)	2	0.0428	2000	0.8429
